# Efficient Use of Graphene Oxide in Layered Cement Mortar

**DOI:** 10.3390/ma15062181

**Published:** 2022-03-16

**Authors:** Shuangshuang Liu, Fenglei Lu, Ya Chen, Biqin Dong, Hongxiu Du, Xiangyu Li

**Affiliations:** 1 College of Civil Engineering, Taiyuan University of Technology, Taiyuan 030024, China; 15661610815@163.com (S.L.); m17836217726@163.com (F.L.); chenya1626@163.com (Y.C.); tydhx@163.com (H.D.); 2 Guangdong Province Key Laboratory of Durability for Marine Civil Engineering, College of Civil and Transportation Engineering, Shenzhen University, Shenzhen 518060, China; incise@szu.edu.cn

**Keywords:** wet casting, graphene oxide, functionally graded materials, chloride ingress

## Abstract

Graphene oxide (GO) has been found to be an attractive nanomaterial to improve the properties of cementitious composites. However, the use of GO in the industry is limited by its high cost. To achieve a higher cost/performance ratio, GO can be strategically applied in certain parts of cementitious composites structure according to the principle of functionally graded materials. In this study, graded distribution of GO in cement mortar was achieved by sequentially casting a fresh GO-incorporated cement layer on another cement mortar layer. The mechanical properties, especially flexural strength, of layered cement mortar were found to be dependent on the GO content, the delay time, and the interface formed due to layering fabrication. With the GO incorporated in the tensile region only (30% of the total depth), the flexural strength of the layered beam attained 90.91% of that of the beam, with GO uniformly distributed throughout the sample. Based on the results of rapid chloride migration tests, when 12 mm GO-incorporated cement mortar layer was used, the chloride migration coefficient was reduced by 21.45%. It was also found that the measured chloride migration coefficient of layered cement mortar agreed with the series model. The present investigation provides an efficient approach to use GO in cement-based materials from the perspective of mechanical and durability properties.

## 1. Introduction

Because of its high compressive strength and low costs, concrete is the most widely used human-made material worldwide. However, as a brittle material, concrete has a low tensile strength (only approximately 10% of its compressive strength [1]) and weak corrosion resistance. In addition, a significant amount of CO_2_ is emitted during the production of Portland cement, an essential constituent of concrete. Therefore, it is imperative to use concrete more efficiently by improving its durability and mechanical properties, particularly tensile strength.

In recent years, the development of nanomaterials has provided valuable opportunities to improve the properties of concrete. Studies have shown that the addition of nanomaterials, such as 0D nanosilica [2,3], 1D carbon nanotubes [4], and 2D graphene oxide (GO) [5,6], can improve the strength, toughness, and durability of cement composites to varying degrees. Compared to 0D and 1D nanomaterials, GO has a significantly larger specific surface area, which helps facilitate its interactions with cement hydration products and provides a physical barrier in an aggressive environment [7]. Zeng et al. [8] demonstrated that GO nanosheets with large aspect ratios are more conducive to reducing both porosity and permeability.

Research [9,10,11,12] on the potential applications of GO in cementitious composites was conducted extensively to improve the mechanical and durability performance. Pan et al. [10] found that 0.05 wt.% GO increased the compressive and flexural strengths of GO-cement by 15%–33% and 41%–59%, respectively. Lu et al. [11] proved that cement with a stabilized GO-Polycarboxylic acid exhibited higher flexural and compressive strengths owing to the better dispersion of GO in the hardened cement matrix. Tong et al. [13] observed that GO could improve the strength and corrosion resistance of cement mortar. Devi et al. [14] concluded that by adding a small amount of GO, durability could be greatly improved for concrete incorporated with 100% recycled concrete aggregate. Indukuri et al. [15] showed that the flexural and compressive strengths of GO cement composites increased by 77.70% and 47.61%, respectively. In addition, the water sorption capacity, chloride migration coefficient, and chloride penetration depth of cement composite specimens with 0.03 wt.% GO decreased by 14.5%, 30%, and 40.47%, respectively. Typically, cement composite members are cast using a single homogeneous mix in which GO is uniformly and homogeneously blended with cement. However, considering its higher costs, applications of GO in the building industry are limited.

In order to achieve higher performance/ratio, GO can be strategically used in certain regions of cement composite members for improving both mechanical and durability properties. For example, GO can be used in the tensile region of flexural members to enhance the crack resistance and thus improve the mechanical properties. This is termed as the graded distribution of GO in a single member and is the principle of functionally graded concrete (FGC), which utilizes multiple mixes in a single element by spatially organizing different mixes. Shen et al. [16] found that the flexural behavior of a four-layered concrete beam with gradually distributed polyvinyl alcohol fibers could be significantly improved compared to its single-layered counterpart. Wen et al. [17] reported that the use of FGC effectively reduced material costs without significantly decreasing the corrosion resistance of chloride ions. Choudhary et al. [18] showed that functionally graded rubberized concrete showed better mechanical and durability properties as compared to rubber fiber concrete with rubber fiber uniformly distributed in the concrete. Recently, FGC received renewed attention as a method of developing more environmentally sensitive concrete structures [19,20,21]. Functionally graded cement composite members can be fabricated by intentionally incorporating GO into specific parts. Therefore, we can reduce the material cost without compromising the mechanical and durability properties. However, to the best of the authors’ knowledge, no research to address the graded distribution of GO in cement composites has been reported to date. In the present work, graded distribution of GO was achieved by using a layering production process. Mechanical and durability properties of the layered samples were then investigated.

Usually, the production process of functionally graded cement composites follows strict requirements. It should be noted that 3D printing [22,23] and multimix spraying [24] are innovative alternatives for fabricating functionally graded cement composites. Currently, if formwork is utilized, various methods ranging from wet-on-hard casting to wet-on-wet casting [25] can be used to produce functionally graded cement composites. The wet-on-hard process involves casting a fresh layer on top of a hardened layer to maintain the interfacial geometry between the two layers as intended. In contrast, the wet-on-wet process involves casting a fresh cement mix layer on another previously placed fresh layer after a short delay time into a single element. The advantages of the wet-on-wet process over the wet-on-hard process are better adhesion between layers owing to cement hydration and intermixing. However, strict requirements for the rheological and thixotropic properties of fresh cement composites must satisfy specified hardened-state performance requirements [26]. Geng et al. [27] investigated the effects of rheological properties, deposition time interval, and interface humidity on the dual-hierarchical structures and mechanical properties of the layer interface. Torelli et al. [28] applied a wet-on-wet process to produce layered cement elements and showed that the relationship between the delay time and layer-to-layer bonding strength is nonlinear. Brault and Lees [29] found that suitable concrete mix combinations must be achieved to satisfy the intended layer-to-layer geometry of horizontally layered multiple mix elements. It was reported [30] that an extended delay time negatively impacted the mechanical shear strength of layered self-compacting concrete fabricated by the horizontally sequential casting of two layers. Therefore, a shorter delay time is beneficial for improving layer-to-layer adhesion by inducing cement hydration across layers [28]. However, the challenge is the deviation of the intended interfacial geometry owing to the fluid property of fresh cement composites.

In this study, a wet-on-wet layering fabrication process was adopted to produce layered cement mortar samples with a graded distribution of GO. The manufacturing process involved casting a fresh cement mortar layer containing GO on top of a previously cast cement mortar layer without GO. The fabrication process was investigated by evaluating the thickness of the layer with GO, delay time, and their effects on the interfacial geometry. The mechanical and durability properties of the layered cement mortar, including the flexural strength, compressive strength, and chloride migration coefficient, were then assessed. Based on the mechanical properties, the optimal thickness of the GO layer was determined.

## 2. Experimental

### 2.1. Materials

Ordinary Portland cement (OPC) of grade P.O. 42.5 manufactured by Zhihai Concrete Co., Ltd. (Taiyuan, China) and ISO standard sand produced by China ISO Sand Co., Ltd. (Xiamen, China) were used. A GO suspension with a concentration of 4 mg/mL was purchased from Graphenea (Spain). GO typically consists of C and O, as listed in Table 1. The major functional groups on the GO surface were –OH and –COOH. The average hydrodynamic diameter of the GO nanosheets was approximately 847.3 nm [31].

A polycarboxylic-based superplasticizer (SP) was used in this study to modify the workability of the cement samples. It was reported that SP could disperse GO in simulated pore solution effectively, based on ultraviolet–visible spectroscopy [33]. It was also experimentally demonstrated that SP did not influence the strength.

### 2.2. Preparation of GO Incorporated Cement Mortar

In this study, two types of cement mortar were prepared. One is plain cement mortar M without GO addition; the other one is GO incorporated cement mortar MGn, in which n refers to GO dosage. For example, MG2 refers to cement mortar samples containing 0.02% GO by weight of cement. For both types of samples, the sand-to-cement and water-to-cement ratios by mass were 2 and 0.4, respectively. The amount of superplasticizer was adjusted to ensure the samples had similar workability. The workability was characterized by using a mini-slump test [34].

Li et al. [35] developed a mixing procedure for MGn sample by first blending a GO aqueous solution with sand and then with cement to enhance the GO dispersion and improve the microstructure of the interfacial transition zone (ITZ) in cement mortar. This approach was adopted in the current study.

After the cement mortar was produced, the fresh mortar mixture was cast into steel molds and vibrated on a vibration table to ensure good compaction. The molds were covered with thin polyethylene sheets to prevent water evaporation. After 24 h, the samples were demolded and cured in a saturated limewater bath at 20 °C until testing.

In the following, MG4 and M were used to fabricate a two-layer cement mortar beam using MG4 in the tensile region. Both mechanical and durability properties were tested in the current study. Flexural and compressive strengths of the layered cement mortar samples were tested. The broken parts of samples after mechanical tests were collected to study the microstructure for samples with and without GO. For durability property, both layered and nonlayered cylindrical samples were prepared to test the chloride diffusion coefficients, which is an important indicator for the durability property of cement-based materials. We used MG4 to prepare the layered sample because it showed the best mechanical properties according to a preliminary study.

### 2.3. Fabrication of Layered Cement Mortar Beams with Graded Distribution of GO

**Figure 1:**
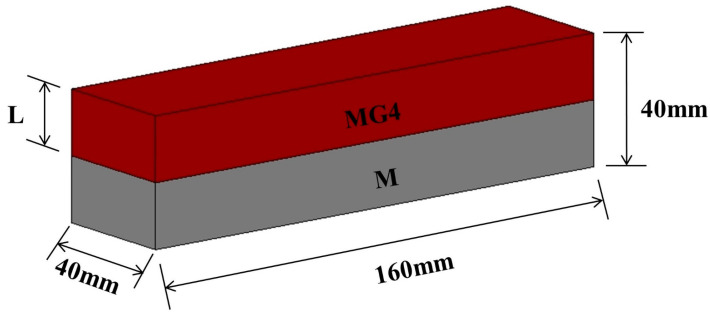
Geometry of layered cement mortar specimens.

Layered cement mortar beams with a graded distribution of GO were fabricated by casting one layer of fresh cement mortar with GO (MG4) on top of another previously cast cement mortar layer without GO (M). Figure 1 shows a schematic of the two-layer beam specimen with dimensions of 40 mm × 40 mm × 160 mm. Two variables were considered in this experiment: the thickness of the MG4 layer and the delay time between two successive castings. Three thicknesses (8, 12, and 16 mm) were selected to evaluate the effect of the MG4 layer thickness on the properties of the layered sample. In addition, 10 delay times were employed to investigate their influence on the properties of the layered samples. Therefore, the layered samples were denoted as L*a*T*b*, in which *a* refers to the thickness of the MG4 layer and *b* the delay time. Because the thickness of the MG4 layer was always shorter than that of the M layer, the M layer was cast first on the bottom to reduce the unfavorable disturbance caused by gravity to the horizontal interface. Table 2 lists all the samples with different MG4 layer thicknesses and delay times.

The samples were fabricated as follows. First, a fresh mixture M with a specified thickness (Table 2) was poured into steel molds of 40 mm × 40 mm × 160 mm. Next, the M layer was vibrated on a vibration table to remove air bubbles trapped inside and placed in an ambient environment for the specified delay time (Table 2). A fresh mixture of MG4 was then slowly deposited on top of the M layer by casting uniformly from one side to the other. The overall sample vibrated again. The process was the same and consistent for all the samples. After the samples were cast, the molds were covered with a thin polyethylene sheet to prevent moisture from escaping and then cured in an indoor environment at 20 °C and relative humidity of 95%. After 24 h, the samples were demolded and cured in a saturated limewater bath at 20 °C until testing.

At 28 days, the flexural and compressive strengths of each group of layered cement mortar beams were determined. The samples were placed correctly to ensure that the MG4 layer was located in the tensile region of the beam. The testing procedures for the mechanical properties were the same as those described in Section 2.2.

### 2.4. Mechanical Tests

Compression and three-point bending tests on the hardened specimens were performed to examine the mechanical properties of the cement mortar samples. For the compression tests, cube specimens of 40 mm × 40 mm × 40 mm were used, and the compressive strength was determined according to ASTM C109. The specimens were subjected to compression tests at 28 days at a loading rate of 0.2 mm/min, corresponding to approximately 0.3 MPa/s. For the three-point bending tests, specimens of 40 mm × 40 mm × 160 mm were used, and the flexural strength was determined following ASTM C78/C78M-10. The specimens were assessed at 28 days at a loading rate of 0.06 MPa/s. The mechanical tests were performed using a servo-hydraulic universal testing machine (SHT4605, MTS, Shanghai, China) with a 600 kN capacity. For both types of tests, at least three duplicate samples were used for each test.

### 2.5. SEM

The microstructure of cement mortar samples was studied by using a field emission SEM (LYRA3, TESCAN, Brno, Czech Republic). SEM samples were collected from central parts of broken samples after compression tests. In order to make the samples conductive, a thin layer of gold-palladium was deposited onto the samples.

### 2.6. Rapid Chloride Migration (RCM) Test

The RCM test method is widely used for determining the chloride migration coefficient of concrete. In the current study, RCM tests on the 28-day cylinder specimens were conducted to assess the chloride migration in the layered cement mortar. For comparison, normal cement mortar samples without layered fabrication were also prepared. Table 3 lists the mix proportions of the layered cement mortar samples and normal samples used for the RCM tests. Cylindrical samples, each with a diameter of 100 mm and a thickness of 50 mm, were used for the tests. For layered samples, they were prepared by casting two cement mortar layers with a delay time of 50 min. Three thicknesses of the layer with GO were used: 8, 12, and 16 mm. For the tests, MG4 was used to prepare layered cement mortar samples to observe the effect of the GO content and layered fabrication on chloride migration. The fabrication of the normal samples (M, MG2, MG4, and MG6) was the same as that described in Section 2.2.

Before the RCM tests, the samples were treated with a vacuum water-saturated machine to fill the internal pores of the samples with an aqueous solution [36]. After 24 h, the chloride migration coefficient (D_RCM_) values of the saturated samples were measured. During the tests, with the cement mortar layer with GO facing the cathode solution, the chloride migration in the mortar was accelerated by applying a constant direct-current voltage (30 ± 0.2 V) between the two circular surfaces of each cylindrical cement mortar sample. After the chloride migration tests, each sample was cut into two halves and sprayed with a 0.1 mol/L AgNO_3_ solution (colorimetric indicator for chlorides) to determine the chloride penetration depth [37].

## 3. Results and Discussions

### 3.1. Microstructure of Cement Mortar Samples

**Figure 2:**
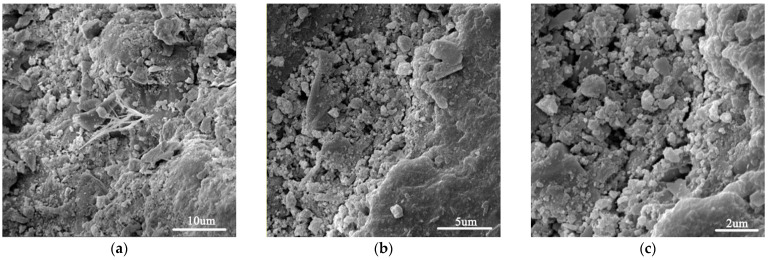
SEM images of plain cement mortar M (**a**–**c**) at different scales.

**Figure 3:**
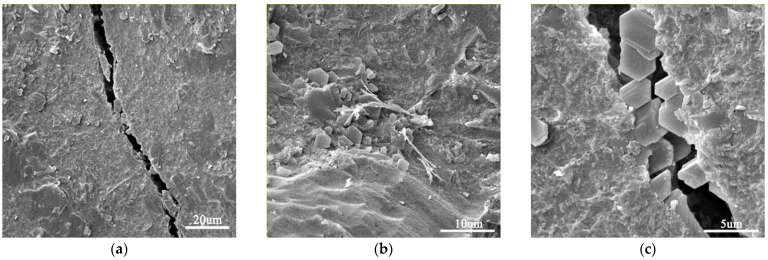
SEM images of GO-incorporated cement mortar MG4 (**a**–**c**) at different scales.

Figure 2 shows SEM images of the plain cement mortar sample. As can be seen, the microstructure was loose and porous. Figure 3 shows images of a GO-incorporated cement mortar sample after a fracture. As can be seen, GO-incorporated cement mortar showed a much denser microstructure. In addition, it can be seen in Figure 3c that a “trans-particle fracture” [38] appears with the crack passing directly through CH crystals, which indicates that C–S–H gels are stronger in GO-incorporated cement mortar.

**Figure 4:**
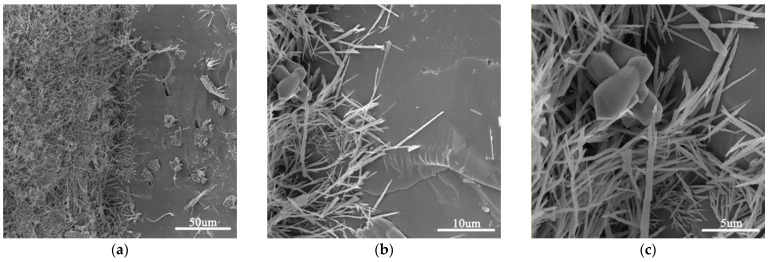
SEM images of an ITZ in sample MG4 (**a**–**c**) at different scales.

Figure 4 shows SEM images of an ITZ in GO incorporated cement mortar. As shown in the figure, the ITZ is quite compact, and precipitated CH crystal was hardly found. There were fibrillar C–S–H and an ettringite network adhering to the surface of a sand grain. This demonstrated that GO addition could improve the microstructure of ITZ, which was also reported by the literature [35]. Denser ITZ is good for improving chloride migration resistance.

### 3.2. Observations of Layer-to-Layer Interface

**Figure 5:**
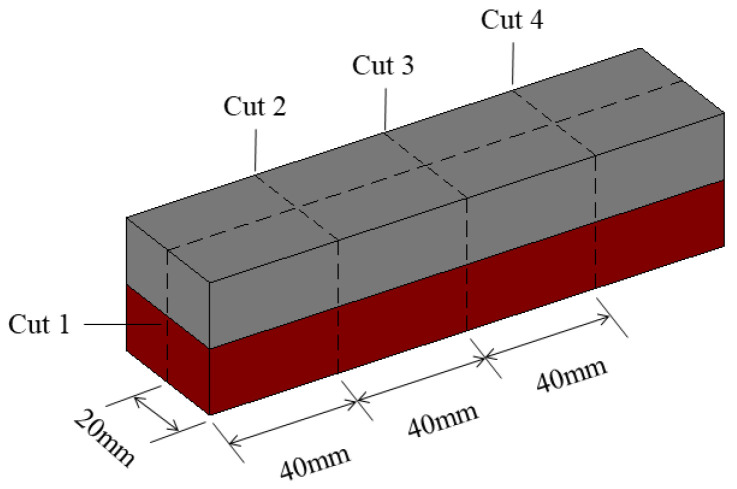
Four cut locations of each sample.

The hardened samples were cut along four planes after casting for 7 days, as shown in Figure 5, to examine the final shape of the samples. Cuts 1 and 3 were the vertical planes in the longitudinal and lateral axes of the center of each sample, respectively. Cut 2 was a transverse vertical plane 40 mm (a quarter of the total length of the sample) from one end of the sample, whereas Cut 4 was at the same distance (40 mm) from the other end of the sample.

**Figure 6:**
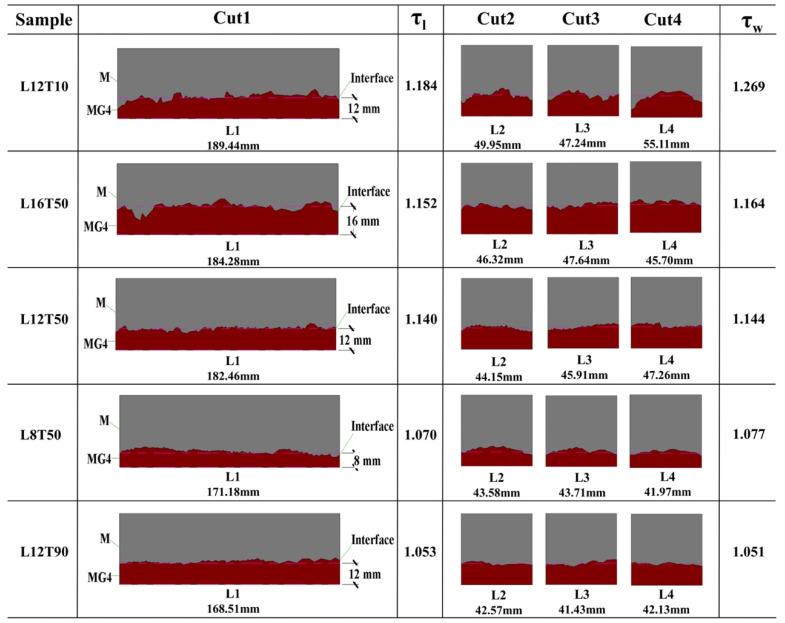
Digital images of each cut of specimens.

Five samples (L12T10, L8T50, L12T50, L16T50, and L12T90) were cut to observe the layer-to-layer interface and analyze the delay time effects on the interfacial surface between two layers. Each cut face was converted into a digital image using a method similar to that used by Torelli and Lees [28]. First, the images of these cross-sections were captured using a high-resolution camera. In addition, the focal plane was parallel to the cuts to ensure that the upstream perspective distortions were minimized. Next, in the raster graphics editor, the residual deformations were corrected by marking several reference points on the cross-section of each sample. Finally, the corrected photographs were imported into the software [39] to trace the outline of the interface between the two mortar layers. Figure 6 shows the digital images of cut sections of five samples and contour lengths in the longitudinal and lateral axes.

When the delay time was 10 min (e.g., L12T10), rough interface boundaries were observed owing to the high fluidity and thixotropy of both the top and bottom mortar layers. When the delay time was 90 min (e.g., L12T90), visually smooth interface boundaries were observed owing to the reduced thixotropy of the bottom layer. When the delay time was 50 min (e.g., L12T50), the roughness of the interface boundary was between those of L12T10 and L12T90. The tortuosity was used in order to characterize the roughness of the interface in both length and width directions, denoted as τ_l_ and τ_w_, respectively. The tortuosity can be calculated by dividing contour length with the sample dimensions (length or width). As presented in Figure 6, L12T10 and L12T90 had contour lengths of 189.44 and 168.51 mm, respectively. Then the tortuosities can be calculated as 1.184 (189.44/160) and 1.093 (168.51/160), respectively. It can be inferred that the delay time played a role in determining the roughness of the interface boundary. When the delay time was 50 min, the interface boundary became rougher with an increase in the gravity of the top layer, as shown in Figure 6 (e.g., L8T50, L12T50, and L16T50). The tortuosities increased with increasing MG4 layer thickness for samples L8T50, L12T50, and L16T50 (Figure 6). From a mechanical point of view, it is desirable to increase the thickness of the MG4 layer. However, a thicker MG4 layer can lead to more significant interface deviations, which may be unbeneficial for adhesion between the two layers.

Studies [40,41,42] showed that horizontally layered FGC elements with a short delay time (20–60 min) between the deposition of each concrete mix layer could be fabricated. A slight delay (at least 20 min) enables the base layer to have some degree of a thixotropic structure before receiving the concrete layer on top, ensuring that the ideal hardened layer geometry is achieved [29]. Andre et al. [29] observed that concerning the expected deviation from a horizontal interface between mixes, the combination of the lower layer mixture (the highest density and the lowest fluidity) and the upper layer mixture (the lowest density and the highest fluidity) is likely the best-case scenario. For sample L12T50 in this study, the fluidity of the top layer was significantly higher than that of the bottom layer, which hydrated for 50 min, although the densities of the two layers were almost equal.

### 3.3. Flexural and Compressive Strengths

**Figure 7:**
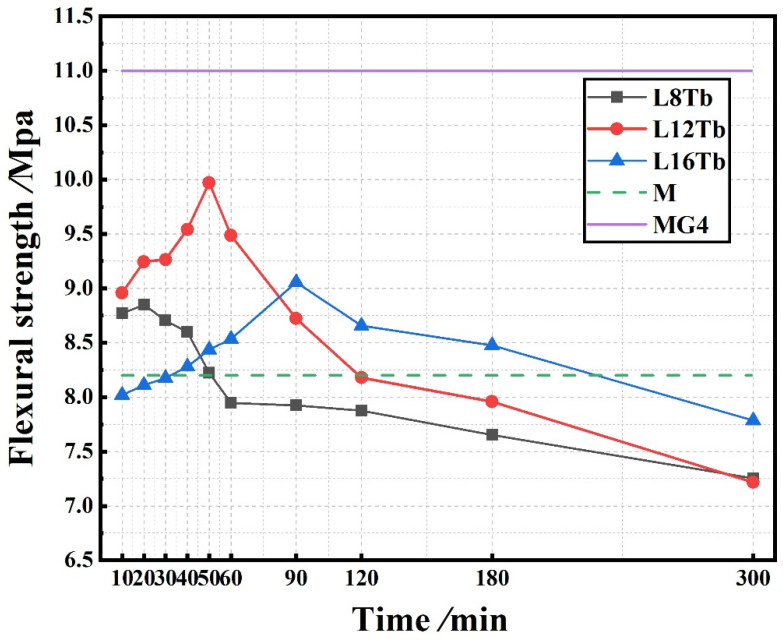
Flexural strength of layered samples.

Figure 7 shows the flexural strength of the layered cement mortar beams. The flexural strength increased to a maximum and then decreased with increasing delay time, regardless of the thickness of the MG4 layer. The flexural strength was the highest for the sample, with an MG4 layer thickness of 12 mm and a delay time of 50 min. For the three types of samples with different MG4 layer thicknesses, the maximum flexural strength was found to be dependent on the delay time. For samples with an MG4 layer thickness of 8 mm, 12 mm, and 16 mm, the optimal delay time was found to be 20 min, 50 min, and 90 min, respectively. When the delay time was longer or shorter than the optimal delay time, the flexural strength was negatively affected. It should be noted that when the second layer (MG4) thickness increased, the optimal delay time also increased. According to the literature [30], the optimal delay time (*T_c_*) can be estimated by using Equation (1), and it can be simplified to Equation (2):

(1) $$T_{c}=\frac{\sqrt{\frac{{\left(\rho gh\right)}^{2}}{12}+{\left(\frac{2\mu_{p}V}{h}\right)}^{2}}}{A_{thix}}$$

(2) $$T_{c}=\frac{\rho gh}{3.5A_{thix}}$$

where ρ and *μ_p_* are the density and viscosity of the top layer at the fresh state, respectively, *V* is the speed of casting, and *A_thix_* is the structuration rate [30] of the top layer.

According to Equation (1), the delay time depends on the thixotropic behavior of MG4, the thickness of the MG4 layer, casting speed, and viscosity of MG4 at the fresh state. Among these parameters, *h* is the only variable since we used fresh MG4 as the top layer for all layered samples. According to Equation (2), the delay time *T_c_* increases with an increase in layer thickness. This is consistent with the results obtained in the current study.

For the layered cement mortar beam with a specific amount of GO, the weakest part of the beam was the interfacial region between two layers if “not immediate” wet-on-wet casting (less than 1 min) was applied. Therefore, the effect of the delay time on the flexural strength could be ascribed to the bonding between the two layers, which formed from the interlocking between two layers at different scales. The optimal delay time was achieved because the bonding could improve when two cement mortar layers contacted each other at a suitable time. Because we cast two layers in the fresh state, the interface was wavy or uneven owing to the plastic and thixotropic behavior of the fresh cement mortar. A suitable uneven interface can enhance the bonding between the two layers owing to mechanical interlocking. However, the interface can be so uneven that the bonding is compromised due to the unwanted stress concentration. The optimal delay time can also be explained from a chemical perspective. The cement hydration products could interlock when two layers were cast in the fresh state because of ion transportation across the interface. When the delay time was very long, the interface was very smooth, and there were hardly any interactions between the hydration products from the two layers, which was unbeneficial for the bonding. In summary, we need to pay special attention to the delay time because the plastic and thixotropic behavior and cement hydration are time-dependent. Thus, it was necessary to establish a more rigorous definition of the allowable setting time, rheological properties, density differentials, and preparation procedure to obtain improved mechanical properties. It should be noted that when the delay time exceeded 90 and 120 min, the flexural strength was lower than that of the plain cement mortar, indicating that GO was no longer useful because the interface was the weakest at this stage.

As shown in Figure 7, sample L12T50, with an MG4 layer of 12 mm and a delay time of 50 min, exhibited maximum flexural strength. The flexural strength of the nonlayered sample without and with GO was 8.2 MPa and 11 MPa, respectively. Compared to the nonlayered sample without GO, the flexural strength of L12T50 increased by approximately 21.57%. The flexural strength of L12T50 was 90.91% of that of the nonlayered sample (MG4 beam sample) containing GO distributed throughout the sample. The MG4 layer thickness was 30% of the total depth of the beam sample. This result indicated that by applying a functionally graded distribution of GO, the GO dosage could be reduced by 70% without compromising the flexural strength. Zhang et al. [43] simulated the flexural performance of layered ECC-concrete composite beams with a fracture mechanics model. When the ECC thickness reached a critical value (30% of the beam height), it was found that the ductility of the member showed a very rapid increase. Guan et al. [44] investigated the mechanical properties of layered concrete beams reinforced with steel fibers distributed in the tensile region. Similar properties were observed when the layer thickness of the incorporated steel fiber was 30% of the total depth of the sample compared to the sample with uniformly distributed steel fibers, consistent with the results of this study.

For samples with an MG4 layer thickness of 8 mm, the flexural strength was lower than those of the samples with MG4 layer thicknesses of 12 and 16 mm when the delay time was longer than 60 min. For samples with an MG4 layer thickness of 16 mm, the flexural strength was slightly higher than that of samples with 12 mm MG4 layer when the delay time exceeded 90 min. Before a delay time of 90 min, the flexural strengths of samples with an MG4 layer thickness of 12 mm were significantly higher than those of samples with a 16 mm layer.

For GO-incorporated layered cement mortar, the combination of the interfacial bonding and GO content increased the flexural strength. For L8 samples, the interfacial bonding should be better due to less disturbance when the delay time was shorter than 60 min. However, when the delay time increased, the interfacial bonding became worse, while an insufficient GO content could not bear the flexural loads and resist crack initiation and development. Although the L16 samples had a higher GO content, which improved the flexural strength, the interfacial bonding of the L16 samples was not necessarily better because of the unfavorable disturbance caused by the gravity of the thicker layer. During the wet-on-wet process, the vibration and gravity loading induced significant disturbance [16] to the bottom layer, adversely affecting the interface geometry and mechanical properties. When the delay time exceeded 90 min, the layer-to-layer interfaces of the L12 and L16 samples were very smooth because the bottom layers had a significantly extended hydration time. Additionally, there were hardly any interactions between the two layers. Therefore, the effect of the GO content was dominant in this case.

**Figure 8:**
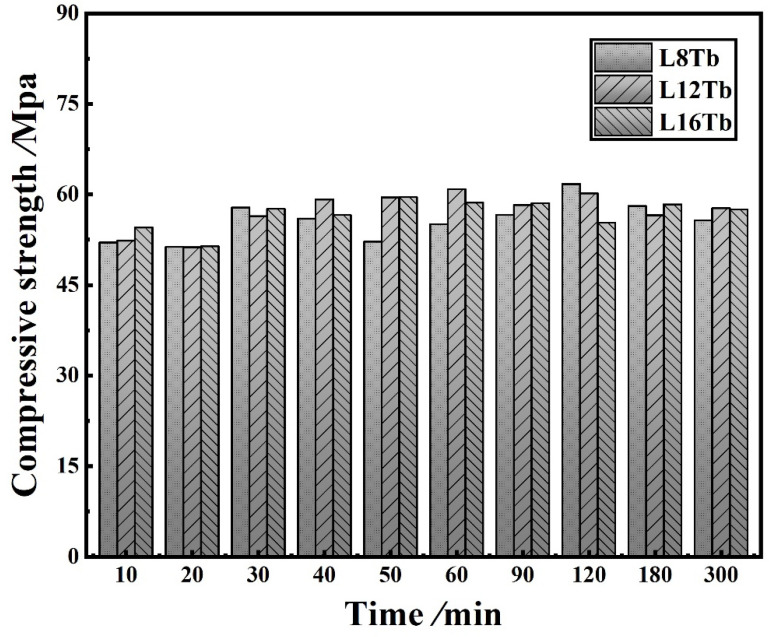
Compressive strength of layered samples.

Figure 8 shows the compressive strength of layered cement mortar samples. There is no clear trend for the compressive strength, although the strength was found to be lower when delay time was shorter than 20 min. Overall, it can be concluded that the graded distribution of GO improved the flexural strength more than the compressive strength.

### 3.4. Flexural Failure Characteristics of Layered Cement Mortar Beams

**Figure 9:**
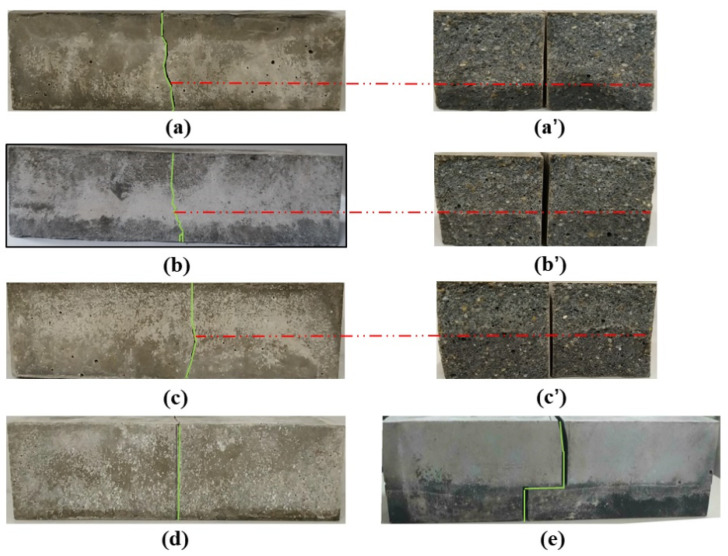
Crack development of samples: (**a**) L8T50, (**b**) L12T50, (**c**) L16T50, (**d**) M, and (**e**) L12T300. Fractured surfaces of samples: (**a’**) L8T50, (**b’**) L12T50, (**c’**) L16T50.

Figure 9 shows the images of the layered cement mortar beams after flexural failure. A significant difference in the crack development between the layered and plain cement mortar beams was observed. For the plain cement mortar (M) beam, the crack was completely vertical, with the fracture surface being relatively flat, as depicted in Figure 9d. However, inclined and tortuous cracks appeared on the layered cement mortar beam containing GO. The cracks obliquely developed until they reached the layer interface, and deflection occurred at the interface. The cracks then continued to propagate obliquely until the specimen fractured entirely. Compared to the fracture surface of the plain sample, the fracture surface of the layered sample with GO was tortuous and rougher, as shown in Figure 9a–c. For plain cement mortar beams, brittleness caused the appearance of vertical cracks and flat fracture surfaces. However, the cracks could be resisted and deflected by the GO in layered cement mortar samples because of its superior mechanical properties and high aspect ratio. Consequently, tortuous cracks and rougher fracture surfaces were formed on the GO-incorporated layered cement mortar beams.

It should be noted that the weakest part of the layered cement mortar beam was the interface formed between the top and bottom layers. When the adhesion between the two layers was not strong sufficiently, the flexural strength could be impacted negatively. To investigate the interface adhesion problem of the layered beam, we examined a special layered beam, as shown in Figure 9e. The MG4 layer was cast 5 h after placing the M layer. During the flexural tests, visible delamination between the two layers was observed. Delamination occurred because of the weak interface bonding strength owing to the incompatible shrinkage of the two layers and the weak interactions between the two layers caused by the extended delay time [45]. In comparison, enhanced interfacial bonding can be achieved by applying a suitable delay time.

**Figure 10:**
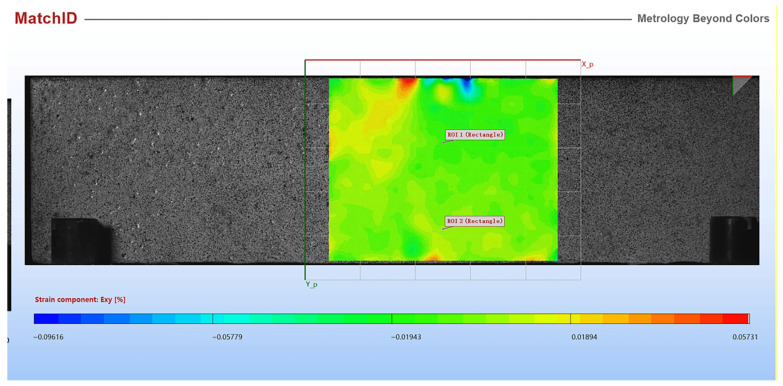

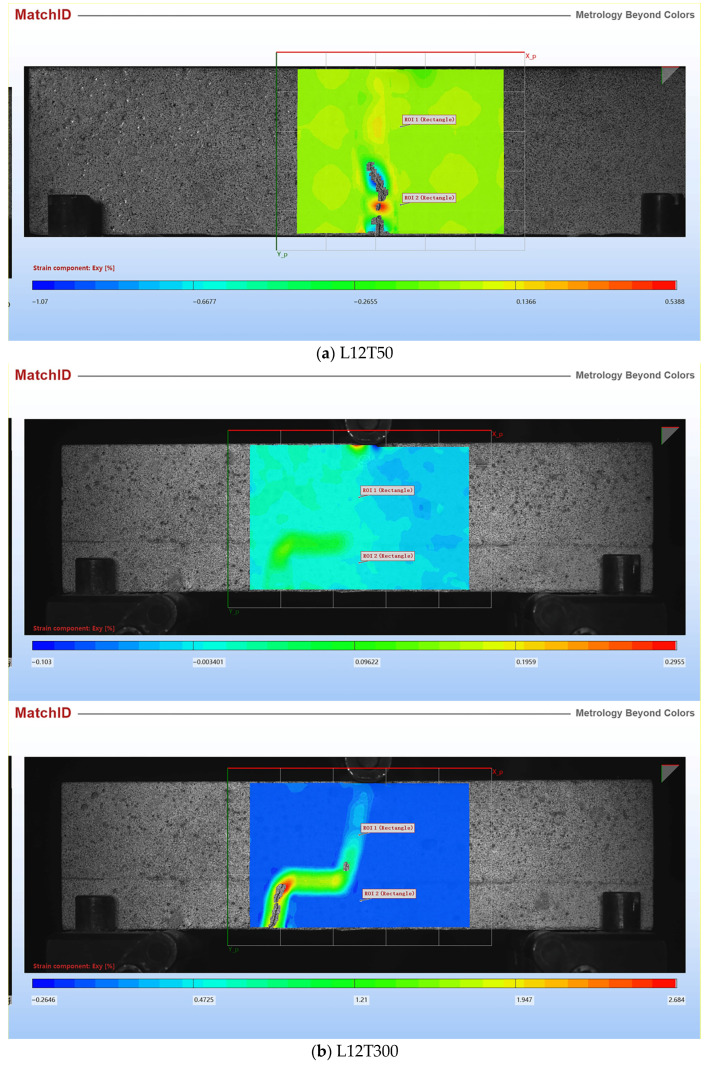
Shear strain distribution of sample L12T50 (**a**) and L12T300 (**b**).

A digital image correlation (DIC) system provided by MatchID (Belgium) was used to measure the deformation field of layered cement mortar samples. The deformation field of a square area located in the middle of the sample was calculated, and then the in-plane shear strain was determined. Figure 10 shows the in-plane shear strain distribution just before failure for samples L12T50 and L12T300. For sample L12T50, there was no sign showing delamination could happen according to shear strain distribution. However, for sample L12T300, it is clear that the shear strain was larger at the interface. Future studies will be conducted to investigate the debonding mechanism of layered cement mortar samples.

### 3.5. Chloride Migration Coefficients of Layered Cement Mortar

**Figure 11:**
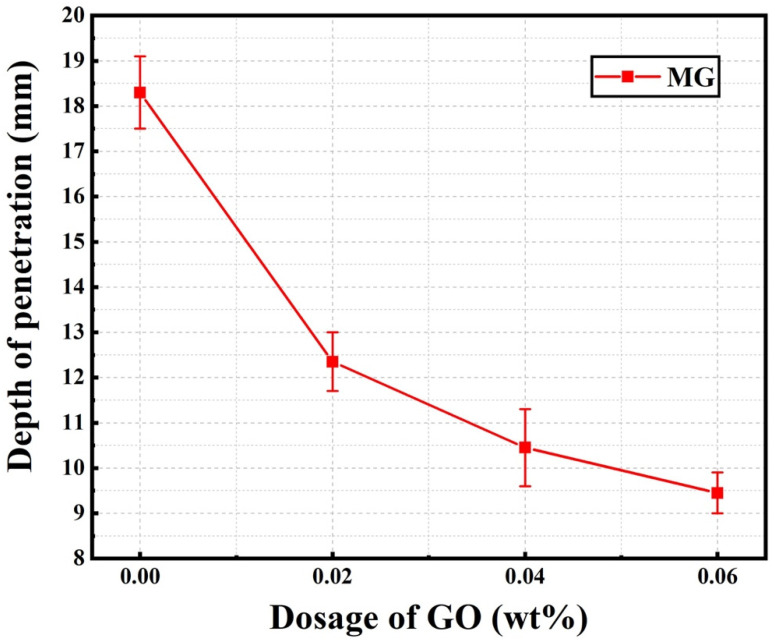
Chloride penetration depth of normal cement mortar samples (M, MG2, MG4, and MG6).

After RCM tests, AgNO_3_ solution was sprayed onto the cut surfaces of the samples. A color change indicated a clear boundary between the chloride-contaminated area (white) and the chloride-free zone (brown) after 15 min. The chloride penetration depth of the normal samples (M, MG2, MG4, and MG6) are shown in Figure 11. The actual heights of the specimens and the penetration depths of chloride ions were measured using a Vernier caliper with an accuracy of 0.1 mm. The chloride penetration depths of MG2, MG4, and MG6 were 12.3 mm, 10.4 mm, and 9.5 mm, respectively, which were 32.5%, 42.9%, and 48.4% lower than that of the plain cement mortar M, respectively, with a penetration depth of 18.3 mm.

After obtaining the parameters from the RCM and chloride penetration depth tests, the chloride migration coefficient (*D_RCM_*) was calculated using the following “traditional” RCM model.

(3) $$D_{RCM}=2.872\times 10^{-6}\frac{Th(x_{d}-\alpha \sqrt{x_{d}})}{t}$$

In Equation (3), *D_RCM_* is the chloride migration coefficient (m^2^/s), *T* is the average value of the initial and final temperatures of the anolyte (K), *h* is the height of the specimen (m), *x_d_* is the average value of chloride penetration depth (m), *t* is the duration of the test (s), and *α* is the auxiliary variable. The auxiliary variable can be calculated using Equation (4).

(4) $$\alpha =3.338\times 10^{-3}\sqrt{Th}$$

**Figure 12:**
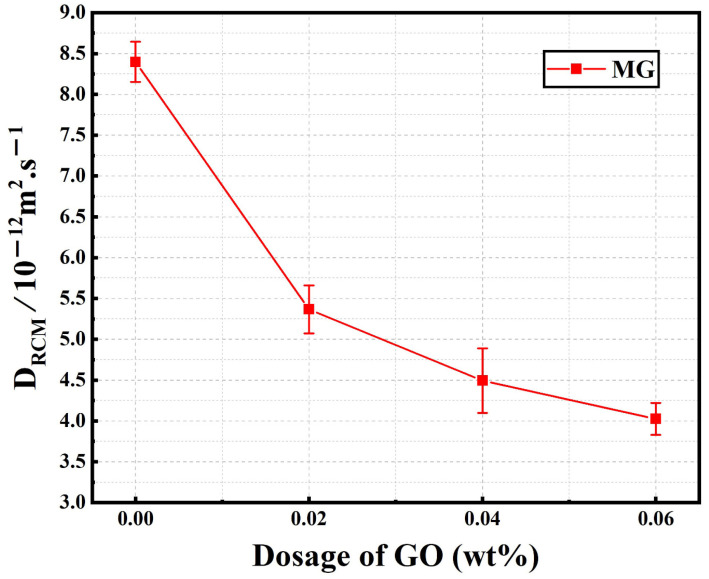
Chloride migration coefficient of normal cement mortar samples (M, MG2, MG4, and MG6).

Figure 12 shows the chloride migration coefficients of the normal samples (MG2, MG4, and MG6) listed in Table 3. As shown in Figure 12, the *D_RCM_* values decreased notably with an increase in GO content. When GO content was 0.04%, the *D_RCM_* value was decreased by 46.5%, compared to plain sample M. This trend indicated that the chloride corrosion resistance of cement mortar could be improved by GO incorporation, which hindered the migration of chloride ions. This inference is in agreement with previous studies [15,46], which concluded that the addition of GO could improve chloride diffusion resistance.

**Figure 13:**
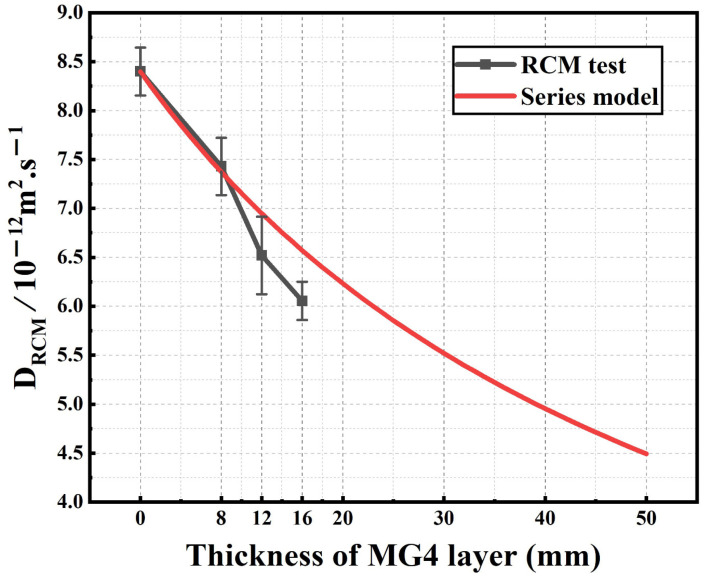
Measured and predicted chloride migration coefficient of layered samples.

Figure 13 shows the chloride migration coefficients of the layered cement mortar samples listed in Table 3. The results showed that the chloride migration coefficient of the samples decreased with an increase in the thickness of the GO-incorporated layer. The chloride migration coefficients of the layered cement mortar sample (L12G4) with 12 mm of MG4 layers decreased by 21.45%, compared to the plain cement mortar sample (M). Although the improvement is not as significant as a normal sample, the material cost from GO was notably reduced. Therefore, more efficient use of GO can be achieved by using it in certain parts of cement-based structural members.

**Figure 14:**
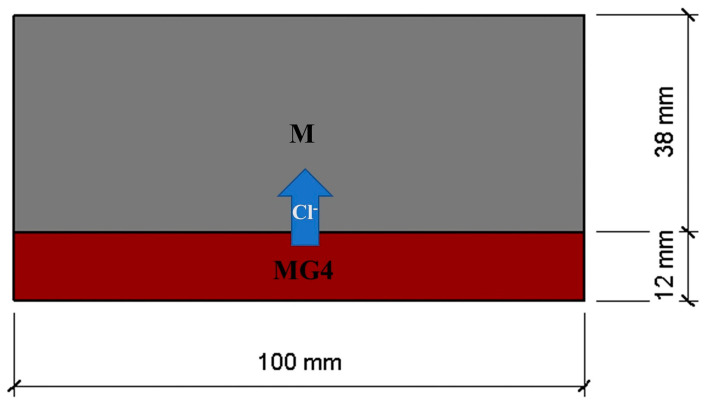
Series model representing layered sample L12T50.

As shown in Figure 14, in the RCM test, the layered sample can be considered as two layers of materials are aligned in series, with chloride ions diffusing uniaxially from the MG4 layer to the M layer. Therefore, the bulk chloride migration coefficient of the layered sample can be calculated as

(5) $$D_{RCM}={\left(\frac{V_{MG4}}{D_{RCM,MG4}}+\frac{V_{M}}{D_{RCM,M}}\right)}^{-1}\;\;$$

where *D_RCM_* of MG4 and M can be found in Figure 14, *V_MG4_* and *V_M_* are volume percentages of MG4 and M layer in the sample, respectively. Then, the *D_RCM_* of the layered sample can be determined by using Equation (5). As shown in Figure 13, the measured *D_RCM_* of the layered sample agreed with the series model. It should be noted that the series model is an ideal model which does not consider the interface between two layers. By conducting mathematical modeling, Andrade et al. [47] reported that 10 mm thick “skin” may be enough to alter the chloride profile during diffusion. Further study is needed to study the impact of the interface that exists between two layers on chloride diffusion.

Cement mortar is a composite material composed of cement paste, ITZ, and sand. Because sand is impermeable, chloride ingress in cement mortar is influenced by the cement paste and ITZ. It is well known that the pore structure, including the porosity and critical pore size, is critical for transporting chloride ions in cement paste and ITZ. However, it is insufficient to consider only the pore structure in investigating the chloride ingress process. It is rational to introduce tortuosity to understand the process better, as it is directly related to the actual transport pathway of ions in cement-based materials.

Guo et al. [48] applied the effective medium theory and established the relationship between the *D_RCM_* of cement mortar and the tortuosity of cement paste and ITZ using Equation (6):

(6) $$D_{\mathrm{RCM}}=\frac{\delta \cdot D_{f}}{0.518}\cdot \frac{{\left(\nu_{p}+\nu_{ITZ}\right)}^{2}}{{\left(\nu_{p}\tau_{p}+\nu_{ITZ}\tau_{ITZ}\right)}^{2}}=\frac{\delta \cdot D_{f}}{0.518}\cdot \frac{{\left(\nu_{p}+\nu_{ITZ}\right)}^{2}}{\tau_{p}{}^{2}\left(\nu_{p}+0.35\nu_{ITZ}\right)}$$

In Equation (6), *δ* is the constrictivity of the pore network of mortar, *D_f_* is the diffusion coefficient of chloride transported in bulk water, ν*_p_* and ν*_ITZ_* are the volumetric contents of bulk cement paste and ITZ in hardened mortar, respectively, and *τ_p_* and *τ_ITZ_* are the tortuosity parameters of the cement paste and ITZ, respectively. The chloride migration coefficient decreased with an increase in the tortuosity parameters of the cement paste and ITZ. Because the cement paste had a significantly higher volume fraction than the ITZ, the tortuosity of the cement paste was more effective in slowing down the overall chloride migration.

The addition of GO as a filler material in cement mortar can introduce a more tortuous path and then increase the tortuosity of the cement paste because it can act as a physical barrier that chloride ions cannot penetrate. The effect of GO on the tortuosity can be defined using Equation (7) [49]:

(7) $$\tau =\frac{d^{\prime }}{d}=1+\frac{L}{2W}\varphi_{s}$$

where *L* and *W* are the characteristic length and thickness of the GO, respectively, and *φ_s_* is the volume fraction of the GO. Compared to other fillers (such as spherical and cubic fillers), GO is particularly efficient for increasing the tortuosity of the matrix because of its unique sheet-like geometry with an ultrahigh aspect ratio (*L/W*).

In addition to increasing the tortuosity of the cement matrix, the incorporation of GO refined the pore structure, improving the chloride resistance of the cement mortar. In particular, the capillary pores affected the transport properties of cement composites because they could provide transport routes for aggressive substances [50]. Mohammed et al. [51] reported that the incorporation of GO in cement mortar effectively resisted chloride ion ingress by refining the pore sizes.

It was reported [52] that total carbonation of CH and C–S–H leads to a complete loss of chloride binding capacity of the cement matrix. However, GO was proved to be effective in inhibiting long-term carbonation of CH and C–S–H [53]. Moreover, from a chemical perspective, Hou et al. [54] investigated the interactions between chloride ions and cement hydrates modified using GO nanosheets through molecular dynamics simulations. The simulations showed that the GO adsorbed on the inner surface of the C–S–H pore could immobilize chloride ions and water, thereby limiting their migration through chemical bonding. Therefore, GO-incorporated cement materials are expected to perform much better during the long-term service period, which cannot be observed in RCM tests conducted in the current study.

According to the present work, as compared to the plain sample, both mechanical and durability properties can be improved for layered cement mortar with GO incorporating in the region subjected to higher tensile stress and aggressive chloride ions. In addition to GO dosage, the production process, especially the delay time, can impact both mechanical and durability properties. The optimal delay time is dependent on the time-varied rheological and thixotropic properties of the cement mortar. In a future study, a detailed time-varying rheological behavior of GO incorporated cement mortar should be investigated to establish a theoretical framework to guide the production process of cement-based materials with a graded distribution of GO by using the wet-on-wet layering production method.

## 4. Conclusions

In this study, functionally graded cement mortar beams were fabricated by casting GO-incorporated cement mortar layers on previously placed cement mortar layers without GO. The thickness of each GO-incorporated cement mortar layer and the delay time was investigated to assess their effects on the mechanical and durability properties. The following conclusions were drawn.

(1) The graded distribution of GO in cement composites could be achieved by casting a fresh GO-incorporated cement layer on another layer. For samples with a specified thickness of the GO-incorporated cement layer, the optimal delay time between sequential castings was determined by analyzing the flexural strength;
(2) When the GO was added in the tensile region only, the mechanical properties of the layered cement mortar beams were not compromised compared to those of the control sample. However, the interface formed between the layers significantly influenced the mechanical properties. Measures to improve interfacial adhesion should be investigated in future studies;
(3) The RCM test results showed that a small amount of GO could significantly slow down chloride ingress. This effect was more pronounced when the dosage or aspect ratio of the GO was increased. The mechanism was that, as a physical barrier, GO nanosheets increased the tortuosity of the cement matrix and immobilized the migration of water and chloride ions through chemical bonding.

This study proved that nanomaterial GO could be strategically applied in parts of cementitious composites based on the principle of functionally graded materials. This approach provides a new perspective for the efficient use of nanomaterials in cement-based materials.

## Figures and Tables

**Table 1:** Elemental analysis of GO [32].

Element	Carbon	Oxygen	Hydrogen	Nitrogen	Sulfur
%	49–56	41–50	0–1	0–1	0–2

**Table 2:** Designations of layered cement mortar samples.

Delay Time (min)	Thickness of MG4 Layer (8 mm)	Thickness of MG4 Layer (12 mm)	Thickness of MG4 Layer (16 mm)
10	L8T10	L12T10	L16T10
20	L8T20	L12T20	L16T20
30	L8T30	L12T30	L16T30
40	L8T40	L12T40	L16T40
50	L8T50	L12T50	L16T50
60	L8T60	L12T60	L16T60
90	L8T90	L12T90	L16T90
120	L8T120	L12T120	L16T120
180	L8T180	L12T180	L16T180
300	L8T300	L12T300	L16T300

**Table 3:** Dry mix compositions of layered and nonlayered cement mortars for RCM tests.

Sample	Water/OPC (by Mass)	Sand/OPC (by Mass)	Thickness of MG (mm)	Delay Time (min)	GO/OPC (%)	SP/OPC (%)
M	0.4	2	/	/	0	0.11
L8T50	0.4	2	8	50	0.04	0.15
L12T50	0.4	2	12	50	0.04	0.15
L16T50	0.4	2	16	50	0.04	0.15
MG2	0.4	2	50	/	0.02	0.13
MG4	0.4	2	50	/	0.04	0.15
MG6	0.4	2	50	/	0.06	0.21

## Data Availability

Data sharing is not applicable to this article.
